# A Novel Method for Early Gear Pitting Fault Diagnosis Using Stacked SAE and GBRBM

**DOI:** 10.3390/s19040758

**Published:** 2019-02-13

**Authors:** Jialin Li, Xueyi Li, David He, Yongzhi Qu

**Affiliations:** 1 School of Mechanical Engineering and Automation, Northeastern University, Shenyang 110000, China; jialinli_neu@163.com (J.L.); lixueyineu@gmail.com (X.L.); 2 Department of Mechanical and Industrial Engineering, The University of Illinois at Chicago, Chicago, IL 60607, USA; 3 School of Mechanical and Electronic Engineering, Wuhan University of Technology, Wuhan 430000, China; yongzhiqu@hotmail.com

**Keywords:** early gear pitting fault diagnosis, vibration signals, SAE, GBRBM

## Abstract

Research on data-driven fault diagnosis methods has received much attention in recent years. The deep belief network (DBN) is a commonly used deep learning method for fault diagnosis. In the past, when people used DBN to diagnose gear pitting faults, it was found that the diagnosis result was not good with continuous time domain vibration signals as direct inputs into DBN. Therefore, most researchers extracted features from time domain vibration signals as inputs into DBN. However, it is desirable to use raw vibration signals as direct inputs to achieve good fault diagnosis results. Therefore, this paper proposes a novel method by stacking spare autoencoder (SAE) and Gauss-Binary restricted Boltzmann machine (GBRBM) for early gear pitting faults diagnosis with raw vibration signals as direct inputs. The SAE layer is used to compress the raw vibration data and the GBRBM layer is used to effectively process continuous time domain vibration signals. Vibration signals of seven early gear pitting faults collected from a gear test rig are used to validate the proposed method. The validation results show that the proposed method maintains a good diagnosis performance under different working conditions and gives higher diagnosis accuracy compared to other traditional methods.

## 1. Introduction

Gears play an important role in mechanical transmission systems. It is necessary to diagnose gear faults to ensure stable and reliable operation of the systems. The methods of fault diagnosis can be roughly divided into two categories: model-driven methods and data-driven methods [1]. Model-based diagnostic methods require a deep understanding of the systems, and many parameter adjustments need to be performed to build the model. Therefore, this paper applies data-driven methods to diagnose gear faults. The data-driven diagnostic process involves two steps: (1) establish a data model based on known state data, (2) use the established model to diagnose mechanical faults. The fault diagnosis process can be regarded as the process of applying the model for pattern recognition. When building a fault diagnosis model, there are generally two processes: feature extraction and pattern recognition [2]. The purpose of feature extraction is to convert high-dimensional data into low-dimensional features, which can better perform pattern recognition. There are many methods for feature extraction such as statistical analysis methods, fast Fourier transform (FFT), Hilbert–Huang transform (HHT) [3], empirical mode decomposition (EMD) [4], wavelet transform (WT) [5], principal components analysis (PCA) [6], and so on. There are many traditional pattern recognition methods including Bayesian classifier [7], K-nearest neighbor (KNN) algorithms [8], artificial neural network (ANN), support vector machine (SVM) [9], etc. Traditional ANN can only distinguish less complex features, and the diagnosis results are greatly affected by process of feature extraction and feature selection. In recent years, research on deep learning is getting popular. Deep learning can improve the shortcomings of traditional neural network. There are many types of deep learning methods applied in fault diagnosis, which can be divided into supervised methods such as deep neural network (DNN), convolutional neural network (CNN) [10] and unsupervised methods such as deep belief network (DBN), and autoencoder (AE).

Zhang et al. [11] used DNN to diagnose bearing faults and directly used the collected vibration signals as the inputs of neural network, removing the error caused by the feature extraction process. Tested by two publicly available data from University of Cincinnati Center for Intelligent Maintenance System (IMS) and Case Western Reserve University (CWRU), their proposed method was shown to be able to effectively diagnose bearing faults. Chen et al. [12] used a CNN to diagnose gearbox faults. FFT was performed on the vibration signals. Statistical methods were used to extract features from the time-domain and the frequency domain signals as inputs to the CNN. Chen et al. [13] applied ensemble empirical mode decomposition (EEMD) to extract features, and then applied DBN to classify the gear faults. Wang et al. [14] applied unsupervised continuous sparse autoencoder (CSAE) for feature learning and connected a layer of back propagation (BP) networks behind the CSAE. The training process first applied CSAE for unsupervised classification, and then used BP for supervised learning.

DBN has been used for fault diagnosis. The research using DBN for fault diagnosis is reviewed next. Tran et al. [15] used Teager–Kaiser energy operator (TKEO) and DBN to diagnose reciprocating compressor valves faults. In their paper, TKEO was proposed to estimate the amplitude envelopes. The collected vibration signal was processed by WT denoising, and then the time domain signal was converted into a frequency domain. Finally, the statistical methods were used to extract the feature as inputs of the DBN. The diagnostic method used by Han et al. [16] was similar to the method in reference [15], except that it adds a particle swarm optimization-support vector machine (PSO-SVM) to classify extracted parameters. Shao et al. [17] applied the dual-tree complex wavelet packet for feature extraction, and then used statistical methods for feature selection. Finally, an adaptive DBN was used for fault classification. Wang et al. [18] also applied statistical methods to process time-frequency domain signals, and then used DBN to detect multiple faults in axial piston pumps. Lee et al. [19] used DBN to diagnose the air handling unit (AHU). Ahmed et al. [20] combined DBN and softmax classifiers to diagnose rolling bearing faults. Tao et al. [21], He et al. [22], and Chen et al. [23] also applied statistical methods for feature extraction, and then applied DBN for fault classification.

Deutsch et al. [24] integrates DBN and a particle filter for bearing remaining useful life (RUL) prediction. Geng et al. [25] were inspired by the glial chains to improve the structure of the restricted Boltzmann machines (RBMs). An improved greedy layer-wise learning algorithm was used to improve the diagnostic accuracy. Ren et al. [26] combined deep belief networks and multiple models (DBN-MMs) to diagnose complex systems faults. Shao et al. [27] combined the CNN with the DBN to process the compressed sensing (CS). In addition, exponential moving average (EMA) technique was used to improve diagnostic accuracy of the constructed deep model. Jiang et al. [28] proposed a feature fusion DBN method to diagnose rotating machinery fault. Moreover, the locality preserving projection (LPP) was used to fusion deep features to further improve the quality of the deep features.

SAE has been used for fault diagnosis recently. The research using SAE to diagnose faults is reviewed next. Shao et al. [29] proposed ensemble deep auto-encoders (EDAEs) to diagnose bearing faults. The effects of different activation functions and various AEs on diagnostic results were discussed in the article. Maurya et al. [30] used stacked autoencoder to fuse the low-level feature. And then a multi-class SVM was used as classifier. Shao et al. [31] applied deep autoencoder to diagnose rotating machinery faults. The maximum cross entropy was used as the loss function and artificial fish swarm (AFS) algorithm was applied to optimize the key parameters of the deep autoencoder. Meng et al. [32] used denoising autoencoder to diagnose bearing faults. They improved the fault diagnosis rate by reusing the data points between the adjacent samples. The hyper parameter was adjusted by changing the number of units per layer to adapt to the different resilience of the layer.

This paper proposed integrates SAE with GBRBM to diagnose early gear pitting faults. The SAE is used to convert high-dimensional data into low-dimensional data, and GBRBM is used to accommodate the continuous distribution of the inputs. The rest of this paper is organized as follows. In Section 2, the proposed method based the stacked SAE and GBRBM is explained in details. In Section 3, the description of the experimental test rig used for collecting the vibration data for the seven gear pitting faults is provided. In Section 4, the validation results and the discussion of the validation results are presented. Finally, Section 5 concludes the paper.

## 2. The Proposed Method

### 2.1. Framework of Proposed Method

**Figure 1:**
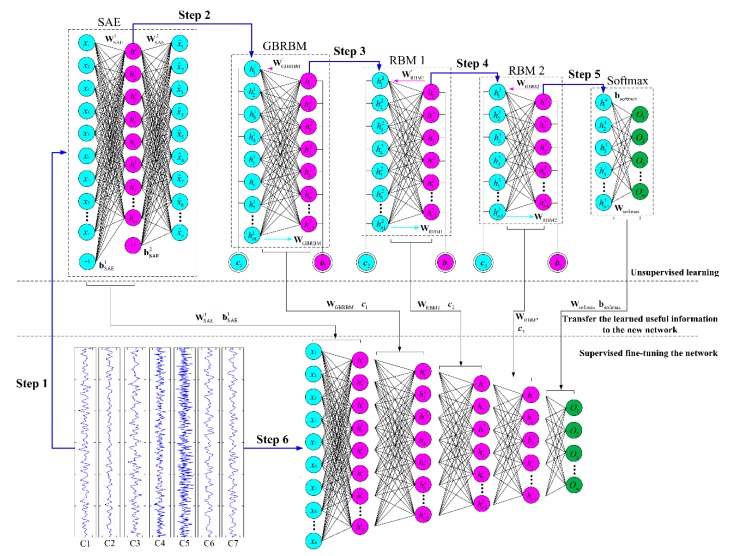
The framework of the proposed method.

Most of the data-driven diagnosis methods involve separate manual feature extraction process. Manual feature extraction mostly relies on human expertise, and the manual feature extraction process is time-consuming and labor intensive. Moreover, the diagnostic results are greatly affected by the feature extraction method. Therefore, diagnostic methods that do not include separate manual feature process are more desirable. Inspired by the unsupervised learning process, this paper proposes a diagnostic method that combines supervised learning with unsupervised learning. The framework of the diagnostic method is shown in Figure 1.

As shown in Figure 1, the framework of the proposed method includes three parts: (1) unsupervised feature learning, (2) transfer the learned useful information to the new network, (3) supervised fine-tuning the restructured network. Stacked SAE, GBRBM and RBMs are combined to work as a simultaneous signal processing and unsupervised feature extraction process. The blue circles in the figure represent the input layer neurons, the red circles represent the hidden layer neurons, and the green circles represent the output layer neurons. The entire diagnostic model has a total of 6 layers of neurons. The specific training process contains 6 steps as shown in Figure 1, raw vibration signals are first used for feature extraction through unsupervised learning, and the data is forwarded through the SAE, GBRBM, two-layer RBM and softmax layers, then fine-tune the weights and biases from unsupervised learning process of each layer according to the cross entropy error function.

The network training in Figure 1 consists of 6 steps. Table 1 shows the detailed calculation principle for the 6 steps, and also includes input values, output values, and parameters transferred for each layer. Figure 1 and Table 1 in combination gives a general understanding of the training procedure. First, the unsupervised learning is performed layer by layer. Then, the learned useful information is transferred into the new network. Finally, supervised fine-tune is performed to adjust the entire network. The detailed equations are shown in Table 1.

### 2.2. Spare Autoencoder

Sparse autoencoder (SAE) [33,34] is an unsupervised learning network mainly used for data dimensionality reduction and feature extraction. The SAE includes three layers: input layer (*n* + 1 neuron), hidden layer (*m* + 1 neuron, *m* < *n*), and output layer (*n* neurons). Figure 1 show the structure of SAE, which can be seen to contain two processes of encoding and decoding.

The encoding process of SAE can be implemented by Equation (1), and the decoding process can be implemented by Equation (2).

(1) $$h=sigm\left(W_{1}x+b_{1}\right)$$

(2) $$\hat{x}=sigm\left(W_{2}h+b_{2}\right)$$

where **x** is the input matrix, **W**_1_ and **b**_1_ are the weight matrix and bias vector between input layer and hidden layer, **h** is the hidden matrix, **W**_2_ and **b**_2_ are the weight matrix and bias vector between hidden layer and output layer, and $\hat{x}$ is the output matrix; function *sigm* (·)=1/(1 + *e*^−z^).

When the mean square error (MSE) is used as the loss function of SAE, the expected processing results usually cannot be achieved. In order to make SAE perform better, a new loss function is designed as Equation (3), which consists of three parts: *J*_MSE_, *J*_weight_, and *J*_sparse_ [35]. The purpose of using *J*_weight_ is to control the value of the connected weights to avoid overfitting [36]. The added *J*_sparse_ is a sparsity penalty term, which can make SAE learn more features from the input by forcing SAE to maintain a degree of sparsity [37,38].

(3) $$J_{\mathrm{SAE}}=J_{\mathrm{MSE}}+\lambda \cdot J_{\mathrm{weight}}+\beta \cdot J_{\mathrm{sparse}}$$

where *J*_MSE_ is the mean square error term as show in Equation (4), *J*_weight_ is the weight penalty item as show in Equation (5), *J*_sparse_ is the sparsity penalty term as show in Equation (6), *λ* is the regularization parameter of weight term, and *β* is the coefficient of sparsity penalty term.

(4) $$J_{\mathrm{MSE}}=\frac{1}{2s}\overset{s}{\underset{i=1}{\sum }}\|x_{i}-{\hat{x}}_{i}\|$$

(5) $$J_{\mathrm{weight}}=\frac{1}{2}\sum_{l=1}^{k-1}\sum_{j=1}^{n_{l}}\sum_{i=1}^{n_{l-1}}W_{ij}^{l}$$

(6) $$J_{\mathrm{spare}}=\sum_{j=1}^{m}\mathrm{KL}(\rho \|{\hat{\rho }}_{j})=\sum_{j=1}^{m}\left(\rho \log\left(\frac{\rho }{{\hat{\rho }}_{j}}\right)+\left(1-\rho \right)\log\left(\frac{1-\rho }{1-{\hat{\rho }}_{j}}\right)\right)$$

(7) $${\hat{\rho }}_{j}=\frac{1}{s}{\overset{s}{\underset{i=1}{\sum }}h}_{ij}$$

where *s* is the sample size of training set, *k* is the number of layers in the network, *n_l_* is the neurons in layer *l*, *ρ* is the set neuron sparsity parameter, and ${\hat{\rho }}_{j}$ is the sparsity of the *j*-th neuron as show in Equation (7).

### 2.3. Develop the GBRBM based on RBM

Restricted Boltzmann Machine (RBM) is the basic component of the deep belief network (DBN) [39,40]. Similar to the SAE, it is also an unsupervised learning network that can be used for feature extraction. The RBM contains two layers: visible layer (contains *n* visible units) and hidden layer (contains *m* hidden units). The neurons in the same layer are not connected, and neurons in different layer are connected in each other. The weight matrix connecting the two layers is denoted by **W**, the bias vector of the visible layer is denoted by **c**, and the bias vector of the hidden layer is denoted by **b**.

Inspired by statistical physics, it can be found that any probability distribution can be transformed into an energy-based model. The joint probability distribution of the visible layer and the hidden layer is proportional to the energy equation [41], as shown in Equation (8). And the joint probability distribution of v and h can be obtained as shown in Equation (9).

(8) $$-\log P\left(v,h\right)\propto E\left(v,h|\theta \right)=-\sum_{i=1}^{n}c_{i}v_{i}-\sum_{j=1}^{m}b_{j}h_{j}-\sum_{i=1}^{n}\sum_{j=1}^{m}v_{i}w_{ij}h_{j}$$

(9) $$P\left(v,h|\theta \right)=\frac{1}{Z(\theta )}\exp\left(-E\left(v,h|\theta \right)\right)$$

where *v_i_* is the visible layer unit, *h_j_* is the hidden layer unit, *w_ij_* is the weights between visible layer and hidden layer, *c_i_* and *b_j_* are the bias of two layers; *m* hidden units in hidden layer, *n* visible units in visible layers, *θ* = {*w_ij_*, *c_i_*, *b_j_*} are the parameters of RBM, and $Z\left(\theta \right)=\sum_{n}\sum_{m}\exp\left(-E\left(v,h|\theta \right)\right)$ is a partition function.

The probability function of the visible layer is given by Equation (10).

(10) $$\begin{array}{ll} P\left(v;\theta \right) & =\sum_{h}P\left(v,h;\theta \right)\\ & =\frac{1}{Z(\theta )}\sum_{h}\exp\left(\sum_{i=1}^{n}c_{i}v_{i}+\sum_{j=1}^{m}b_{j}h_{j}+\sum_{i=1}^{n}\sum_{j=1}^{m}v_{i}w_{ij}h_{j}\right)\\ & =\frac{1}{Z(\theta )}\exp\left(\sum_{i=1}^{n}c_{i}v_{i}\right)\times \prod_{j=1}^{m}\sum_{h}\exp\left(b_{j}h_{j}+\sum_{i=1}^{n}v_{i}w_{ij}h_{j}\right) \end{array}$$

Combining Equations (9) and (10), the conditional probability of the hidden layer can be obtained as shown in Equation (11).

(11) $$\begin{array}{ll} P\left(h|v;\theta \right) & =\frac{P\left(v,h;\theta \right)}{P\left(v;\theta \right)}\\ & =\prod_{j}\frac{\exp\left(b_{j}h_{j}+\sum_{i=1}^{n}v_{i}w_{ij}h_{j}\right)}{\sum_{h}\left(b_{j}h_{j}+\sum_{i=1}^{n}v_{i}w_{ij}h_{j}\right)}=\prod_{j}P\left(h_{j}|v\right) \end{array}$$

Similarly, the conditional probability of the visible layer can be based on the joint probability of **v** and **h** divided by independent probability of hidden layer, as show in Equation (12).

(12) $$P\left(v|h;\theta \right)=\frac{P\left(v,h;\theta \right)}{P\left(h;\theta \right)}=\prod_{i}P\left(v_{i}|h\right)$$

The neurons in the same layer are not connected, meaning that the units are conditionally independent. So the conditional probability of the visible layer and hidden layer can be calculated by Equations (13) and (14).

(13) $$P\left(v_{i}=1|h\right)=sigm\left(c_{i}+\sum_{j}w_{ij}h_{j}\right)$$

(14) $$P\left(h_{j}=1|v\right)=sigm\left(b_{j}+\sum_{i}v_{i}w_{ij}\right)$$

where sigm(x)=1/(1+exp(−x)) is the sigmoid function.

The parameter update of the RBM can be obtained by performing a stochastic gradient descent on the negative log-likelihood probability of the training data. The gradient of the negative log probability visible layer to the network parameters can be calculated by Equations (15)–(17). The value of <·>data is easy to get, but the value of <·>model is difficult to get. Therefore, the contrastive divergence (CD) algorithm was proposed by Hinton [42].

(15) $$\frac{\partial \log p(v;\theta )}{\partial w_{ij}}=(<v_{i}h_{j}{>}_{\mathrm{data}}-<v_{i}h_{j}{>}_{\mathrm{model}})$$

(16) $$\frac{\partial \log p(v;\theta )}{\partial b}=(<h_{j}{>}_{\mathrm{data}}-<h_{j}{>}_{\mathrm{model}})$$

(17) $$\frac{\partial \log p(v;\theta )}{\partial c}=(<v_{i}{>}_{\mathrm{data}}-<v_{i}{>}_{\mathrm{model}})$$

where <·>_data_ indicates expectations for data distribution and <·>_model_ is the expectation of the distribution of the model definition.

Both the visible layer and the hidden layer of RBM are binary layers. It is not appropriate to construct the RBM with the binary visible layer when the input is a continuous valued data. So this paper is to develop the Gauss-Binary RBM (GBRBM) [43,44,45] instead of standard RBM, and the energy function of the standard RBM in Equation (8) is changed to Equation (18).

(18) $$E\left(v,h|\theta \right)=-\sum_{i=1}^{n}\frac{{\left(v_{i}-c_{i}\right)}^{2}}{2\sigma_{i}^{2}}-\sum_{j=1}^{m}b_{j}h_{j}-\sum_{i=1}^{n}\sum_{j=1}^{m}\frac{v_{i}}{\sigma_{i}^{2}}w_{ij}h_{j}$$

where $\sigma_{i}^{2}$ is the variance of Gaussian distribution.

With the energy equation in Equation (18), the conditional probability between the visible layer and the hidden layer can be obtained according to the derivation process in Section 2.2.

(19) $$P\left(v_{i}=v|h\right)=N\left(v;c_{i}+\sum_{j}w_{ij}h_{j},\sigma_{i}^{2}\right)$$

(20) $$P\left(h_{j}=1|v\right)=sigm\left(b_{j}+\sum_{i}\frac{v_{i}}{\sigma_{i}^{2}}w_{ij}\right)$$

where *N*(·, *μ*, $\sigma_{i}^{2}$) is Gaussian distribution, also called normal distribution, *μ* is the mean, and $\sigma_{i}^{2}$ is the variance.

The softmax classification layer is commonly used in the last layer of the neural network, and its working principle is shown in Equations (21) and (22).

(21) $$y_{j}=softmax(\sum_{i=1}^{p}\left(h_{i}w_{ij}+d_{j}\right))$$

(22) $$softmax(z_{i})=e^{z_{j}}/\sum_{j=1}^{q}e^{z_{j}}$$

where *w_ij_* and *d_j_* are weights and bias of softmax layer, *h_i_* is the input of softmax layer, *p* is the number of neurons in input layer, and *q* is the number of neurons in output layer.

## 3. Experiment Setup and Data Acquisition

**Figure 2:**
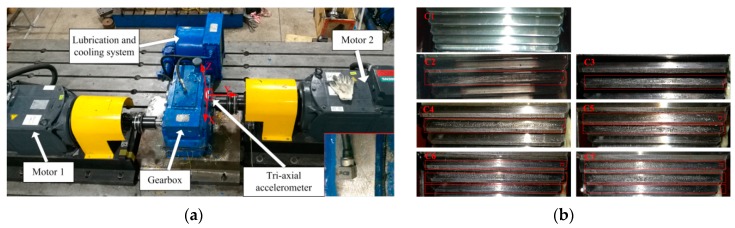
(**a**) Experimental test rig (**b**) gear pitting type.

In this paper, vibration data collected from experiments of seven gears with early gear pitting faults on a gear test rig were used to validate the proposed method. Figure 2 shows the gear test rig and the seven gears with the early gear pitting faults. The gearbox in the test rig consists of a pair of spur gears. The pinion gear is the driving gear (including 40 teeth, module 3 mm), and the large gear is the driven gear (including 72 teeth, module 3 mm). The gearbox is powered by two Siemens servo motors with a power of 45 kW. Motor 1 is the driving motor and motor 2 is the loading motor. The gearbox is equipped with a lubrication and cooling system. The tri-axial acceleration sensor was mounted on the gearbox housing (the red box in the figure) with a sampling rate of 10240 Hz, and the vibration signals in the three directions of X, Y and Z were collected.

The gear pitting faults were artificially manufactured by the drill on the driven gear surface. The specific conditions of the gear pitting faults are shown in Table 2. The fault degree is gradually increased and the latter one fault includes all of the previous fault conditions.

The vibration signals were collected under 25 working conditions. The 25 working conditions included combinations of five speeds (100–500 rpm) and five torque levels (100–500 Nm). Taking the working condition of 500 rpm–500 Nm as an example, each of seven gear types performed five independent data acquisitions and resulted in a total of 35 sets (120,000 data points per set) of data. 80% of all the data was used for training and the remaining data was used for testing. Hence, a training data matrix of 120,000 × 28 and testing data matrix of 120,000 × 7 were generated.

**Figure 3:**
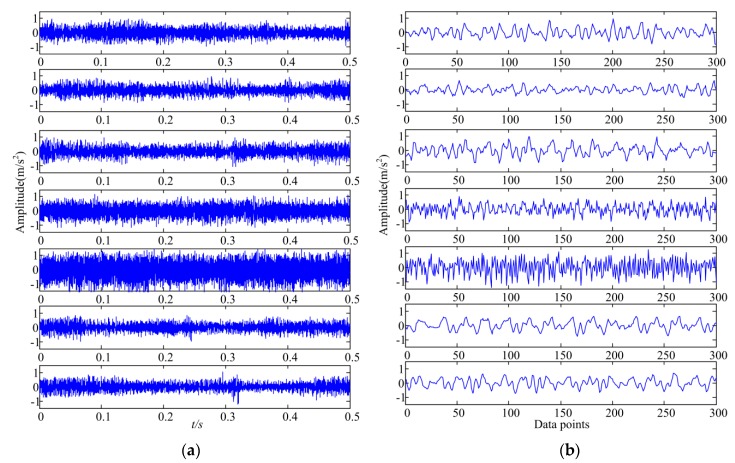
Sample vibration signals of seven gear types in *Z*-axis under 500 rpm–500 Nm: (**a**) signal with length of 0.5 s, (**b**) signal segment containing 300 data points.

If the data matrix is directly used as the inputs, the network will be complex and the training will be slow. Therefore each data set was divided into several segmentations. For the sampling rate of 10240 Hz and a rotation speed of 500 RPM, approximately 1200 data points per gear rotation can be computed. In each segment, 300 data points (quarter of the collected data per gear rotation) were included [46]. In this case, the training data matrix dimension was 300 × 11200 and test data matrix dimension was 300 × 2800. Figure 3a shows sample vibration signals of the seven gears in *Z*-axis under 500 rpm–500 Nm working condition and Figure 3b represents one segment of the corresponding sample vibration signals.

## 4. Results and Discussion

### 4.1. PCA Data Visualization During the Training Process

To show the effectiveness by stacking SAE and GBRBM for extracting useful gear pitting fault information from the raw vibration signals, the network was trained with data from working condition 500 rpm–500 Nm. A total of six layers of neurons constitute the proposed diagnostic model, as shown in Figure 1. The structure of the proposed diagnostic model had the following structure: SAE: 300 × 300 (300 neurons in the input layer and 300 neurons in the hidden layer), GRRBM: 300 × 200 (300 neurons in the visible layer and 200 neurons in the hidden layer), RBM 1: 200 × 100 (200 neurons in the visible layer and 100 neurons in the hidden layer), RBM 2: 100 × 50 (100 neurons in the visible layer and 50 neurons in the hidden layer), Softmax: 50 × 7 (50 neurons in the input layer and seven neurons in the output layer). The size of the weight matrix and the biases were determined by the structure of the proposed model. The initial weights (W1 and W2) of SAE layer were randomly generated between 0 and 1. The initial weights of the softmax layer were randomly generated between 0 and 0.5. The remaining initial weights and biases were set to 0. The proposed diagnostic model was trained layer by layer. Steps 1, 2, 3, 4, and 6 were trained in 300 epochs, respectively. The parameter λ of SAE layer was set to be 0.005, *β* set to 1.5, and *ρ* set to 0.1. The learning rate of GBRBM was set to 0.005, the learning rate of RBM set to 0.5, and the learning rate of the back propagation process set to 0.05. The minimum training error of the back propagation process was set to 0.05. The entire network was calculated on a mini-batch with the batch size set to 100. There are many related parameters affecting the performance of the diagnostic model. The key parameters such as learning rate, structure of the network, and training epochs that have a great impact on the diagnostic results will be discussed in Section 4.3 below.

**Figure 4:**
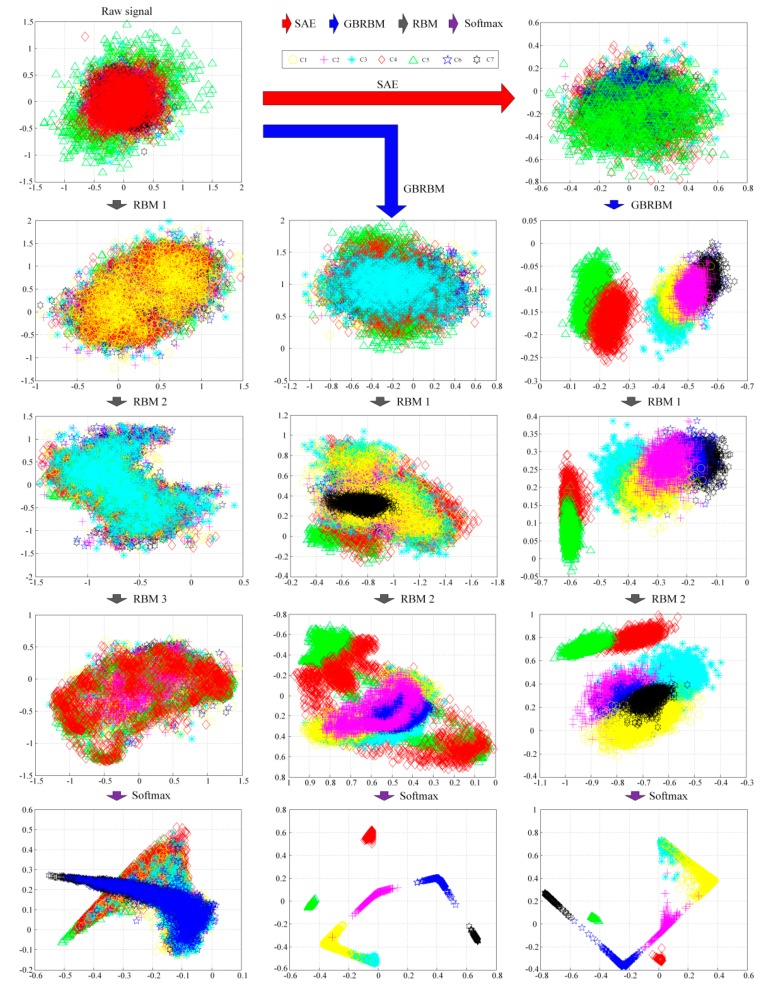
Each layer PCA result of three methods.

The outputs of each layer in the network structure were obtained and these outputs were further processed by PCA. The first two principal components of the PCA results are used to draw a scatter plot in Figure 4 to show the changes of data. The effectiveness of each layer of the network can be judged by observing the changes in the data through each layer of the neural network. In the experiment, the training and testing of the diagnostic model were performed using MATLAB 2014a software. The PCA results shown in Figure 4 were also obtained using the MATLAB codes. All the computational experiments were carried out on a PC with Windows 7 system and a CPU of Intel(R) Core i5-6500 @ 3.2GHz.

**Figure 5:**
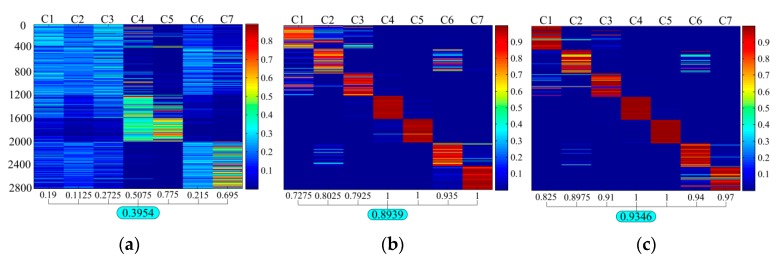
Confusion matrix of the fault diagnosis results: (**a**) standard DBN, (**b**) the first RBM layer of DBN replaced by GBRBM, (**c**) the proposed method.

In Figure 4, three methods are shown. The first column in Figure 4 represents a standard DBN. The middle column represents the method with the first RBM layer of the standard DBN replaced with a GBRBM. The third column represents the proposed method by adding the SAE layer. As can be seen in Figure 4, the proposed method has the best fault separation result, and the separation result of the middle method is better than the standard DBN. Also seen from Figure 4, as the data moves from top down, the level of the fault separation is getting better. Figure 5 shows the confusion matrix of the gear pitting fault diagnosis results of the three methods. Again, as shown in Figure 5, the proposed method has the best diagnosis accuracy of 0.9346, the method with the first RBM layer of the standard DBN replaced with a GBRBM has a diagnosis accuracy of 0.8939, and the standard DBN has the worst accuracy of 0.3954. Even though the confusion matrix shown in Figure 5b looks similar to that in Figure 5c obtained by the proposed method, the diagnostic accuracy for the confusion matrix shown in Figure 5b is 0.8939 while the diagnostic accuracy for the confusion matrix shown in Figure 5c is 0.9346. Therefore, the proposed method gives more accurate diagnosis results. As shown in Figure 5, the graph located at the 2nd row in the middle column represents the PCA result without going through the SAE layer, while the graph located at the 2nd row in the 3^rd^ column represents the PCA result after being processed by the SAE layer. By comparing these two graphs in Figure 4, one should note that the PCA result obtained by the SAE layer in the proposed method gives a better pitting fault separation. The results have shown the effectiveness of SAE layer in the proposed method for extracting useful fault features when it is used for processing the vibration signals.

### 4.2. Diagnostic Results of Proposed Method

**Figure 6:**
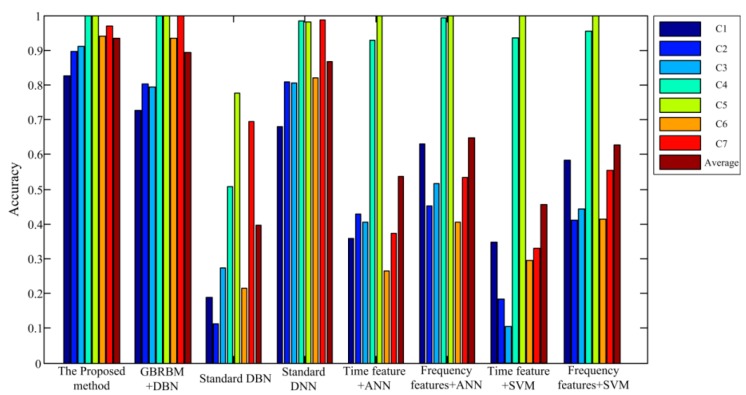
Diagnosis accuracy for all seven gear pitting conditions and the averaged accuracy under 500 rpm–500 Nm working condition.

Figure 6 shows the diagnostic accuracy of the proposed method and the other seven traditional methods. The 7 traditional methods include: (1) The first RBM layer of DBN replaced by GBRBM, (2) standard DBN, (3) standard DNN, (4) ANN with time domain vibration features, (5) ANN with frequency domain vibration features, (6) SVM with time domain vibration features, and (7) SVM with frequency domain vibration features. The results include the diagnostic accuracy for each gear pitting fault condition under 500 rpm–500 Nm working condition and the averaged accuracy over seven gear pitting fault conditions. From Figure 6, in comparison with other methods, the performance of the proposed method is significantly better than other methods. It can also be seen that the diagnostic accuracy for gear pitting conditions C4 and C5 is maintained at a high level in various methods, indicating that they are easier to diagnose than other fault conditions. This can be explained by observing the vibration signal in Figure 3b. It can be found that the vibration signal of C4 and C5 are clearly distinguished from the other gear pitting fault signals.

**Figure 7:**
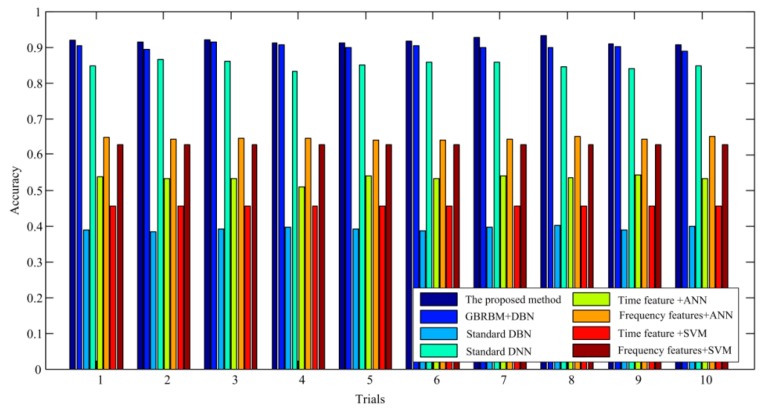
Averaged diagnosis accuracy of the ten trails under 500 rpm–500 Nm working condition.

Figure 7 shows the averaged diagnostic accuracy over all seven gear pitting conditions under 500 rpm–500 Nm working condition in ten trials with eight different methods. It can be seen that the proposed method has the highest diagnostic accuracy. In comparison with the proposed method, the accuracy of the method with the first RBM layer of the standard DBN replaced with a GBRBM is slightly lower. The standard DNN methods also have more prominent diagnosis results.

**Figure 8:**
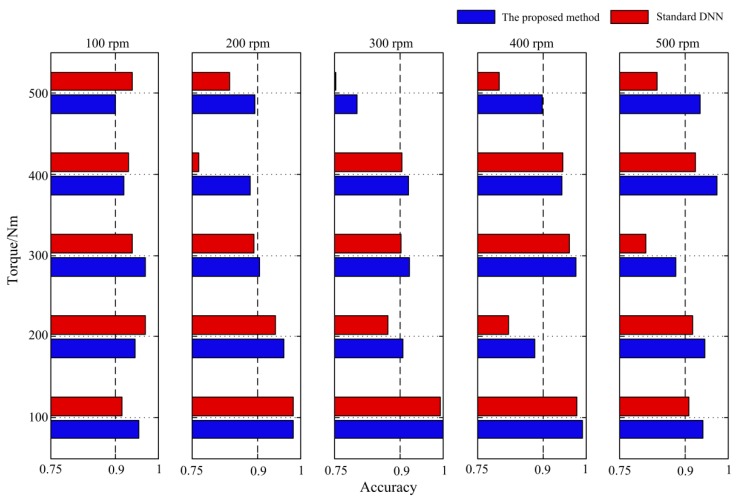
Diagnostic accuracy under 25 working condition.

As shown in Figure 6 and Figure 7, among the methods compared with the proposed method, standard DNN has shown a competitive performance under the 500 rpm–500 Nm working condition. To show the performance of the proposed method in comparison with DNN for all the working conditions, the vibration signals under 25 working conditions were used compute the diagnostic accuracy for both the proposed method and the standard DNN. The results are provided in Figure 8, Table 3 and Table 4.

In Figure 8, the averaged diagnosis accuracy over seven gear pitting conditions under 25 working conditions is provided for both the proposed method and the standard DNN. Further, the average accuracy over all five torque levels for each speed in Figure 8 is computed and provided in Table 3. The average accuracy over all five speeds for each torque level in Figure 8 is computed and provided in Table 4.

It can be seen from Figure 8, Table 3 and Table 4 that the average diagnostic accuracy of the proposed method is higher than that of the standard DNN under various speeds and torque conditions.

It can be seen from Table 3 and Table 4 that the diagnostic accuracy under 100 Nm working condition can reach to 0.9729. In order to prove the repeatability of the diagnosis results, five consecutive diagnoses were performed for five working conditions under 100 Nm. The diagnostic results are shown in Table 5. The averaged diagnostic accuracy of the five diagnosis results under 100Nm working condition is 0.9744, indicating that the proposed diagnostic method has high diagnostic reliability.

### 4.3. The Effect of the Parameters on the Diagnostic Accuracy

To investigate effect of the parameters of the proposed method on the performance of the gear pitting fault diagnosis, experiments were performed. In the first experiment, diagnostic accuracy results with epochs increased from 30 to 300 in an increment of 5 were obtained. In the network structure of the proposed method, the number of neurons in the input layer and the output layer were 300 and 7.

**Figure 9:**
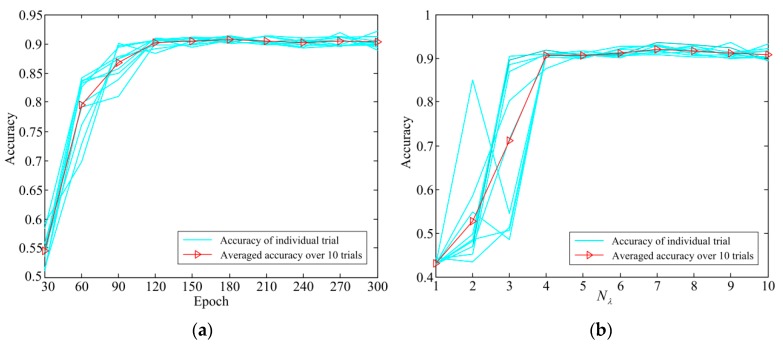
Parameters affecting the diagnosis accuracy: (**a**) epochs, (**b**) *N*_λ._

In order to investigate the impact of the network structure on the performance of the proposed method, a structure parameter Nλ was designed to represent the middle layer. Let Nλ be an integer coefficient between 1 and 10. In this case, the network structure of the proposed method can be represented as: 300-Nλ×(30-20-10-5)-7. In the second experiment, diagnostic accuracy results with Nλ increased from 1 to 10 in an increment of 1 were obtained. The results of the first and second experiments are provided in Figure 9. From Figure 9a, the average accuracy of ten trials gradually increases when the training epochs increased from 30 to 120, and reached to constant level after 120 epochs. Figure 9b shows the effect of the parameter N_λ_ on the diagnostic accuracy of the network structure. When N_λ_ is increased from 1 to 4, the diagnostic accuracy is greatly improved. However, as N_λ_ reaches over 4, the improvement becomes insignificant.

**Figure 10:**
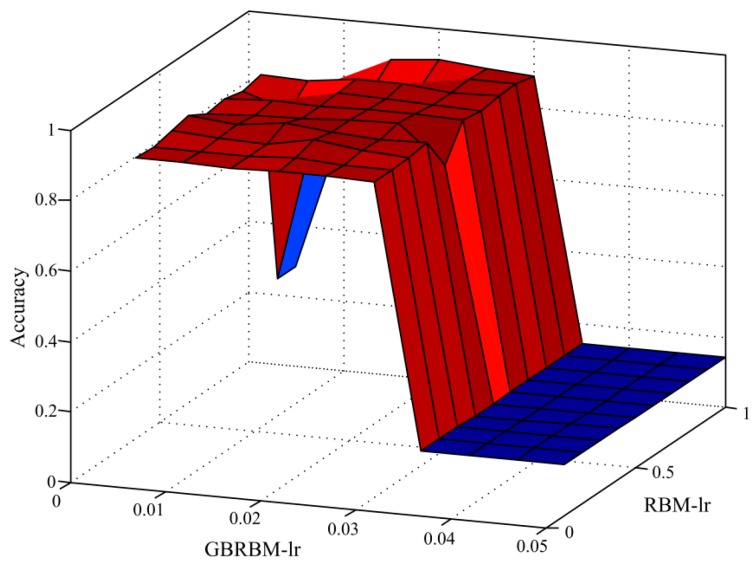
The influence of learning rate on the diagnosis accuracy.

To investigate the impact of the learning rate on the performance of the proposed method, in the third experiment, diagnostic accuracy results with the different learning rates (lr) in RBM and GBRBM were obtained. The results are provided in Figure 10. As seen from Figure 10, the learning rate of GBRBM has a greater impact on the diagnostic accuracy. When the learning rate of GBRBM is greater than 0.03, the accuracy decreased rapidly.

## 5. Conclusions

In this paper, a novel method for early gear pitting fault diagnosis with raw vibration signals as direct inputs was presented. The method was developed by stacking a spare autoencoder (SAE) and a Gauss-Binary restricted Boltzmann machine (GBRBM). The vibration data collected from the gear test rig was used to validate the diagnostic capability of the proposed method. The validation results have shown that the proposed method is capable of gear pitting fault diagnosis with high accuracy. The performance of the proposed method was also compared with other 7 methods including: (1) The first RBM layer of DBN replaced by GBRBM, (2) standard DBN, (3) standard DNN, (4) ANN with time domain vibration features, (5) ANN with frequency domain vibration features, (6) SVM with time domain vibration features, and (7) SVM with frequency domain vibration features. The results of the comparison have shown that the proposed method outperform the other methods in terms of the gear pitting fault diagnostic accuracy. The effect of parameters of the proposed method on the diagnostic performance of the proposed method was investigated and discussed in the paper.

## Figures and Tables

**Table 1:** Detailed process of proposed method.

**Overall process:** (1) Unsupervised: SAE→GBRBM→RBM^(1,2)^→Softmax→(2) Supervised: Back propagation	
**Step 1: SAE training** **Input:** training data **x**, **W** and **b**, *λ*, *β*, *ƞ*_1_, max-epochs^(1)^ **for** *i* to max-epochs^(1)^ $h=sigm\left(W_{1}x+b_{1}\right)$ $\hat{x}=sigm\left(W_{2}x+b_{2}\right)$ $J_{\mathrm{SAE}}=J_{\mathrm{MSE}}+\lambda \cdot J_{\mathrm{weight}}+\beta \cdot J_{\mathrm{sparse}}$ $\Delta w_{ij}^{l}=\Delta_{1}\partial J_{\mathrm{sparse}}/\partial w_{ij}^{l}$, $\Delta b_{i}^{l}=\Delta_{1}\partial J_{\mathrm{sparse}}/\partial b_{i}^{l}$ **end** **Output:** $h_{\mathrm{SAE}}$, $W_{1}^{\mathrm{SAE}}$, $b_{1}^{\mathrm{SAE}}$ **Step 2: GBRBM training** **Input:** $h_{\mathrm{SAE}}$, max-epochs^(2)^, $w_{ij}^{1}$, $c_{i}^{1}$, $b_{j}^{1}$, $\sigma_{i}^{2}$, *ƞ*_2_, *α*_1_ **for** *i* to max-epochs^(2)^ $p(v_{i}=v|h)=N\left(v,c_{i}^{1}+\sum_{j}w_{ij}^{1}\cdot h_{j},\sigma_{i}^{2}\right)$ $p(h_{j}=1|v)=sigm\left(b_{j}^{1}+\sum_{i}\frac{v_{i}}{\sigma_{i}^{2}}w_{ij}^{1}\right)$ $\Delta w_{ij}^{new}=\alpha_{1}\Delta w_{ij}^{1}+\eta_{2}\left(<v_{i}^{\left(0\right)}h_{j}^{\left(0\right)}-v_{i}^{\left(1\right)}h_{j}^{\left(1\right)}>\right)$ $\Delta b_{j}^{new}=\alpha_{1}\Delta b_{j}^{1}+\eta_{2}\left(<h_{j}^{\left(0\right)}-h_{j}^{\left(1\right)}>\right)$ $\Delta c_{i}^{new}=\alpha_{1}\Delta c_{i}^{1}+\eta_{2}\left(<v_{i}^{\left(0\right)}-v_{i}^{\left(1\right)}>\right)$ **end** **Output:** $h_{\mathrm{GBRBM}}$, $W_{\mathrm{GBRBM}}$, $b_{\mathrm{GBRBM}}$ **Step 3: RBM1 training** **Input:** $h_{\mathrm{GBRBM}}$, max-epochs^(3)^, $w_{ij}^{2}$, $c_{i}^{2}$, $b_{j}^{2}$, *ƞ*_3_, *α*_2_ **for** *i* to max-epochs^(3)^ $p(v_{i}=1|h)=sigm\left(c_{i}^{2}+\underset{j}{\sum }w_{ij}^{2}h_{j}\right)$ $p(h_{j}=1|v)=sigm\left(b_{j}^{2}+\underset{i}{\sum }v_{i}w_{ij}^{2}\right)$ The update process of $\Delta w_{ij}^{new}$, $\Delta b_{j}^{new}$ and $\Delta c_{i}^{new}$ is similar to GBRBM **end** **Output:** $h_{\mathrm{RBM}1}$, $W_{\mathrm{RBM}1}$, $b_{\mathrm{RBM}1}$	**Step 4: RBM2 training** **Input:** $h_{\mathrm{RBM}1}$, max-epochs^(4)^, $w_{ij}^{3}$, $c_{i}^{3}$, $b_{j}^{3}$, *ƞ*_4_, *α*_3_ The training process is similar to step 3. **Output:** $h_{\mathrm{RBM}2}$, $W_{\mathrm{RBM}2}$, $b_{\mathrm{RBM}2}$ **Step 5: Softmax layer** **Input:** $h_{\mathrm{RBM}2}$, $w_{ij}^{4}$, $d_{j}$ $y_{j}=softmax\left(\sum_{i=1}^{p}\left(h_{i}w_{ij}^{4}+d_{j}\right)\right)$ $softmax\left(z_{i}\right)=\left(e^{z_{j}}/\sum_{j=1}^{q}e^{z_{j}}\right)$ **Output:** y, $W_{\mathrm{softmax}}$, **d** **Step 6: Back propagation** **Input:** **y**, max-epochs^(5)^, *ƞ*_5_ **for** *i* to max-epochs^(5)^ $W_{1}^{\mathrm{SAE}}$, $b_{1}^{\mathrm{SAE}}$, $W_{\mathrm{GBRBM}}$, $b_{\mathrm{GBRBM}}$, $W_{\mathrm{RBM}1}$, $b_{\mathrm{RBM}1}$, $W_{\mathrm{RBM}2}$, $b_{\mathrm{RBM}2}$, $W_{\mathrm{softmax}}$, **d** as the weight and bias of fully connected DNN. $E_{cross-entropy}=-\left(\left(o\log y\right)+\left(1-o\right)\log\left(1-y\right)\right)$ $\Delta w=\frac{\partial E_{cross-entropy}}{\partial w}$, $\Delta b=\frac{\partial E_{cross-entropy}}{\partial b}$ **end all** **Output: the trained network** **Step 7: Test the trained network with test sample**

**Table 2:** Driven gear pitting type.

Label	Gear Pitting Type		
	72th Tooth	First Tooth	Second Tooth
C1	healthy	healthy	healthy
C2	healthy	10% in middle	healthy
C3	healthy	30% in middle	healthy
C4	healthy	50% in middle	healthy
C5	10% in middle	50% in middle	healthy
C6	10% in middle	50% in middle	10% in middle
C7	30% in middle	50% in middle	10% in middle

**Table 3:** Averaged accuracy under 5 speeds.

Speed	Proposed Method	Standard DNN
100 rpm	**0.9374**	0.9372
200 rpm	**0.9245**	0.8824
300 rpm	**0.9091**	0.8831
400 rpm	**0.9372**	0.9003
500 rpm	**0.9344**	0.8791

**Table 4:** Averaged accuracy under 5 torques.

Torque	Proposed Method	Standard DNN
100 Nm	**0.9729**	0.9546
200 Nm	**0.9279**	0.9036
300 Nm	**0.9294**	0.8997
400 Nm	**0.9275**	0.8935
500 Nm	**0.8848**	0.8307

**Table 5:** Diagnosis accuracy of 5 trials under 5 working conditions.

Working Condition		Trial 1	Trial 2	Trial 3	Trial 4	Trial 5	Row Average
100 Nm	100 rpm-100 Nm	0.9546	0.9554	0.9621	0.9354	0.9843	0.9584
	200 rpm-100 Nm	0.9861	0.9636	0.9736	0.9236	0.9857	0.9665
	300 rpm-100 Nm	1	0.9986	0.9975	0.9989	0.9961	0.9982
	400 rpm-100 Nm	0.9954	0.9968	0.9950	0.9921	0.9961	0.9951
	500 rpm–100 Nm	0.9582	0.9557	0.9446	0.9550	0.9557	0.9539
Column Average		0.9789	0.9740	0.9746	0.9610	0.9836	**0.9744**
